# Association of SNP Rs9943582 in *APLNR* with Left Ventricle Systolic Dysfunction in Patients with Coronary Artery Disease in a Chinese Han GeneID Population

**DOI:** 10.1371/journal.pone.0125926

**Published:** 2015-05-19

**Authors:** Pengyun Wang, Chengqi Xu, Chuchu Wang, Yanxia Wu, Dan Wang, Shanshan Chen, Yuanyuan Zhao, Xiaojing Wang, Sisi Li, Qin Yang, Qiutang Zeng, Xin Tu, Yuhua Liao, Qing K. Wang, Xiang Cheng

**Affiliations:** 1 Laboratory of Cardiovascular Immunology, Institute of Cardiology, Union Hospital, Tongji Medical College, Huazhong University of Science and Technology, Wuhan, P. R. China; 2 Key Laboratory of Molecular Biophysics of the Ministry of Education, College of Life Science and Technology, Center for Human Genome Research and Cardio-X Institute, Huazhong University of Science and Technology, Wuhan, P. R. China; 3 The First Hospital of Wuhan City, Wuhan, P. R. China; 4 Center for Cardiovascular Genetics, Department of Molecular Cardiology, Lerner Research Institute, Cleveland Clinic, and Department of Molecular Medicine, Department of Genetics and Genome Sciences, Case Western Reserve University, Cleveland, United States of America; Scuola Superiore Sant'Anna, ITALY

## Abstract

Heart failure affects 1–2% of the adult population worldwide and coronary artery disease (CAD) is the underlying etiology of heart failure in 70% of the patients. The pathway of apelin and its apelin receptor (APJ) was implicated in the pathogenesis of heart failure in animal models, but a similar role in humans is unknown. We studied a functional variant, rs9943582 (-154G/A), at the 5’-untranslated region, that was associated with decreased expression of the APJ receptor gene (*APLNR*) in a population consisting of 1,751 CAD cases and 1,022 controls. Variant rs9943582 was not associated with CAD, but among CAD patients, it showed significant association with left ventricular systolic dysfunction (431 CAD patients with left ventricular systolic dysfunction (LV ejection fraction or LVEF< 40%) *versus* 1,046 CAD patients without LV systolic dysfunction (LVEF>50%) (*P-adj* = 6.71×10^-5^, OR = 1.43, 95% CI, 1.20–1.70). Moreover, rs9943582 also showed significant association with quantitative echocardiographic parameters, including left ventricular end-diastolic diameter (effect size: increased 1.67±0.43 mm per risk allele A, *P* = 1.15×10^-4^), left atrial size (effect size: increased 2.12±0.61 mm per risk allele A, *P* = 9.56×10^-4^) and LVEF (effect size: decreased 2.59±0.32 percent per risk allele A, *P* = 7.50×10^-15^). Our findings demonstrate that allele A of rs9943582 was significantly associated with left ventricular systolic dysfunction, left ventricular end-diastolic diameter, the left atrial diameter and LVEF in the CAD population, which suggests an important role of the apelin/APJ system in the pathology of heart failure associated with ischemic heart disease.

## Instruction

Heart failure (HF) is an inexorable disease associated with an unacceptably high rate of morbidity and mortality. In developed countries, about 2% of adults suffer from heart failure, and this rate increases to 6–10% in people over the age of 65 years [1]. In China, nearly 10 million people suffer from HF, which is responsible for at least 20 percent of all hospital admissions among people older than 65 years [2].

Left ventricular systolic dysfunction (LVSD) is a complex clinical syndrome that can result from structural or functional cardiac disorders that impair the ability of the ventricle to eject blood [3]. It is the main phenotype of chronic heart failure. Epidemiological studies found that male gender, less education, inactivity, smoking, obesity, diabetes, hypertension, valvular heart disease, coronary artery disease (CAD) and genetics are all independent risk factors for LVSD and heart failure [1,4].

The most common cause of HF is CAD [5,6]. A 13 multicenter trial involving up to 20,000 HF patients reported that CAD was the underlying etiology of HF in nearly 70% of the patients [6]. The loss of contractile ability and cardiac remodeling in patients with CAD are the most important causes for the development of HF, especially its major manifestation of left ventricle systolic dysfunction.

In addition to the classic risk factors such as smoking, hypertension, hypercholesterolemia, diabetes mellitus and obesity, genetic factors also contribute significantly to the development of HF [7,8,9]. Genetic risk factors have been identified for monogenic HF as in the case of inherited hypertrophic cardiomyopathy (HCM) and dilated cardiomyopathy (DCM) and for complex polygenic HF with CAD, myocardial infarction (MI) and hypertension as contributing factors [8]. Many genes have been identified for HCM and DCM, however, genetic analysis of the complex HF, especially HF caused by CAD, needs more investigation.

The apelin receptor (APJ) (encoded by the *angiotensin receptor-like1* gene, *AGTRL1 or APLNR*) is a G-protein-coupled receptor (GPCR), which was identified as the receptor for the adipokine apelin [10,11]. Previous studies showed that apelin activated the APJ pathway through G_i_, and exerted a positive effect on cardiac contractility, and may play an important role in the pathology of HF [12,13]. A recent study on *APLNR* knockout mice showed that APJ receptor could induce a pathological stretch signaling pathway and triggered myocardial hypertrophy under the condition of pressure overloading. The activation of APJ by apelin can blunt the stretch-mediated myocardial hypertrophy. These results showed that the APJ might exert a complex effect on the pathology of HF [14].

To investigate the relationship between the Apelin-APJ pathway and HF in humans, here we analyzed a functional variant, rs9943582, in the promoter region of the *APLNR* gene which encode the APJ receptor in 1,751 CAD patients with different levels of left ventricle systolic function, and evaluated the contribution of the *APLNR* gene to the genetic susceptibility of left ventricle systolic dysfunction in CAD patients.

## Materials and Methods

### Study population

The subjects in this research were from the GeneID population, which is a large ongoing database with clinical data and tissue samples from Chinese patients and controls, and aims to identify susceptible genes for cardiovascular and cerebrovascular diseases in the Chinese Han population [15,16]. The studies were approved by Medical Ethical Committee of Huazhong University of Science and Technology, Ethical Committee of Collage of Life Science and Technology of Huazhong University of Science and Technology and Medical Ethical Committee the First Hospital of Wuhan City and conformed to the guidelines set forth by the Declaration of Helsinki. Written informed consent was obtained from all subjects. All participants are of the ethnic Han origin by self-description.

The study cohort contained 1,751 CAD patients evaluated by coronary angiography. We followed the ACC/AHA criteria, and classified individuals with ≥70% luminal stenosis in at least one main vessel, percutaneous coronary angioplasty, coronary artery bypass graft or MI as CAD cases. MI was defined as typical chest pain of >30 min, characteristic electrocardiographic features of acute MI, and elevation of cardiac enzymes. Subjects with childhood hypertension, congenital heart disease, and type I diabetes mellitus were excluded. Gensini scores were calculated to evaluate the severity of coronary atherosclerosis [17]. We also enrolled a control cohort for CAD from the GeneID database which contained 1,022 samples. The controls were evaluated by coronary angiography and found to have no detectable coronary stenosis (<50%) or history of CAD. However, we were unable to exclude the possibility that some control subjects might have developed CAD after angiographic exams. This issue was partially minimized by the older age of 63.72±8.83 years for controls than that for CAD cases (61.60±9.81 years) (**Table 1**).

**Table 1:** Characteristics of the study population for CAD.

Characteristic	CAD Patients (n = 1,751)	Controls (n = 1,022)	*P* ^†^
Age (years)*	61.60±9.81	63.72±8.83	0.01
Gender, female (%)	39.70%	40.51%	0.67
Hypertension (%)	58.82%	51.37%	1.79×10^–5^
Systolic blood pressure(mmHg)	136±22	126±18	8.84×10^–3^
Diastolic blood pressure(mmHg)	92±15	89±14	0.21
Diabetes (%)	16.80%	12.33%	1.37×10^–3^
Total Cholesterol (mmol/L)	4.41±1.09	4.24±1.09	3.12×10^–3^
Triglyceride (mmol/L)	1.92±1.33	1.81±1.44	0.04
HDL-C (mmol/L)	1.10±0.43	1.20±0.35	0.01
LDL-C (mmol/L)	2.70±1.05	2.42±1.00	4.21×10^–4^
Smoker (%)	33.81%	25.64%	3.67×10^–5^
Gensini score	28.55±23.15	n.a	n.a

Data are shown as mean +/- standard deviation (SD) for quantitative variables and % for qualitative variables.

CAD: coronary artery disease; HDL-c: high density lipoprotein cholesterol levels; LDL-c, low density lipoprotein cholesterol levels; n.a: no data

*Age at the first diagnosis of the disease in CAD cases and age at enrollment for CAD controls.

^†^ *P* value for comparison of means for quantitative data with a student t-test, and for comparison of distribution of qualitative variables between cases and controls with a Chi-square test.

For analysis of the association of rs9943582 with HF in CAD patients, we divided the CAD patients into two sub-groups according to the left ventricle performance by echocardiography, and performed a case-control association analysis. The left ventricle systolic dysfunction (LVSD) sub-group was defined as CAD patients who had a left ventricle ejection fraction (LVEF) of less than 40%, and the normal LVEF sub-group was defined as CAD patients with a LVEF of greater than 50% [18,19]. LVSD patients who had experienced an acute MI within the previous 3 months, a history of significant concomitant diseases, including cardiomyopathies, primary valvular disease, bleeding disorders, renal failure, previous thoracic irradiation therapy, overt infections, or malignant diseases were excluded. In all 1,751 CAD patients, 431 were classified into the LVSD sub-group and 1,046 were divided into the normal LVEF sub-group.

Echocardiographic measurements of left ventricular internal dimension, thicknesses of the interventricular septum, LV thickness of the posterior wall, the diameter of the aortic root (all measured at end-diastole) and the left atrium size at end-systolic were obtained by using a leading edge technique and on average measurements in 3 cardiac cycles according to the American Society of Echocardiography guidelines [20]. Demographic and clinical data such as age, gender, smoking, hypertension, diabetes mellitus and lipid concentrations (including total cholesterol (Tch), triglyceride (TG), high density lipoprotein cholesterol (HDL-c), and low density lipoprotein cholesterol (LDL-c)) were collected. Hypertension was defined as systolic blood pressure of ≧140 mmHg or diastolic blood pressure of ≧90 mmHg and/or ongoing anti-hypertensive treatment of definitively diagnosed hypertension. Type 2 diabetes was diagnosed as features of diabetes with ongoing therapy for diabetes and/or with a plasma glucose level of ≧ 200 mg/dL (11.1 mmol/L), or a fasting plasma glucose concentration of≧126 mg/dL (7.0 mmol/L) after at least 8 hours fasting, or a 2 hours plasma glucose level of ≧200 mg/dL (11.1 mmol/L) during an oral glucose tolerance test (OGTT).

### Genotyping of SNP rs9943582

Genomic DNA was extracted from venous blood samples using the Wizard Genomic DNA Purification Kit (Promega Corporation) following the protocol of the manufacturer.

Genotyping for SNP rs9943582 was performed using a SYTO9 fluorescent dye (Invitrogen Inc) based High Resolution Melt (HRM) method on a Rotor-gene 6200 System (Qiagen Inc) according to the protocol of the manufacturer. In brief, a short fragment containing SNP rs9943582 was amplified with the forward primer of 5’-ACCACTTCCTGCCTGCCCTTTA-3’ and a reverse primer of 5’-ACACCCTCCTTGCTCCCTACCA-3’ in a final concentration of 5μM SYTO9 fluorescent dye. Then the PCR products were genotyped by HRM analysis. DNA samples with known genotypes were used as positive controls, and appropriate negative controls were also included in every genotyping batch to ensure the quality. The quality of genotyping was also ensured by direct DNA sequencing analysis of 50 randomly selected samples (100% consistent rate between the two methods).

### Statistical analysis

Statistical analysis was performed as previously reported [21,22]. Chi-square tests were used to compare the distribution of genotypic or allelic frequencies of the variant and qualitative variables for a case-control association study. Multiple testing was adjusted by Bonferroni correction. Multivariate logistic regression analysis was used to adjust risk factors such as age, gender, smoking, hypertension, diabetes mellitus and lipid concentrations. Empirical *P* values were calculated using 100,000 Monte Carlo simulations. Echocardiographic parameters including LV diastolic internal dimension, LA size, thicknesses of the interventricular septum, LV thickness of the posterior wall, diameter of the aortic root and LVEF were considered as continuous traits and linear regression was used to analyze the association between SNP and these traits. The association was quantified by the regression slope (β), its standard error (SE), and *P* value. The analysis was performed using SPSS version 17.0.

Hardy-Weinberg linkage disequilibrium test was carried out using PLINK. Statistical Power analysis was performed by a free software- PS: Power and Sample Size Calculation [23].

## Results

### Study populations

Clinical characteristics of all study populations are shown in **Tables 1–3**. The population for association analysis between rs9943582 and CAD consisted of 1,751 CAD patients and 1,022 controls and their characteristics are shown in **Table 1**. Among the CAD patients, 431 patients showed LVSD (LVEF<40%), whereas 1,046 patients had normal LVEF (>50%) (**Table 2**). The basic characteristics of these two sub-populations are shown in **Table 2**. We used Gensini scores to evaluate the severity of coronary atherosclerosis of the CAD patients and found no significant differences between the CAD group with LVSD and the CAD group with normal LVEF (*P* = 0.16; **Table 2**). The detailed echocardiographic parameters for the two sub-populations are shown in **Table 3**, and include LV diastolic internal dimension, thicknesses of the interventricular septum, LV thickness of the posterior wall, diameter of the aortic root, left atrium size and LVEF.

**Table 2:** Characteristics of the CAD group with LVSD and the CAD group with normal LVEF.

Characteristic	CAD with LVSD (n = 431)	CAD with normal LVEF (n = 1046)	*P* ^†^
Age (years)*	60.51±11.52	60.30±10.56	*P* = 0.81
Gender, female (%)	39.90%	39.39%	*P* = 0.85
Hypertension (%)	60.56%	58.51%	*P* = 0.34
Systolic blood pressure(mmHg)	135±13	135 ±11	*P* = 0.47
Diastolic blood pressure(mmHg)	90 ±10	86±12	*P* = 0.10
Diabetes (%)	18.09%	16.73%	*P* = 0.61
Total Cholesterol (mmol/L)	4.58±1.28	4.42±1.18	*P* = 0.03
Triglyceride (mmol/L)	1.85±1.28	1.82±1.35	*P* = 0.60
HDL-c (mmol/L)	1.10±0.70	1.10±0.32	*P* = 0.99
LDL-c (mmol/L)	2.86±1.09	2.69±1.07	*P* = 7.12×10^–3^
Smoker (%)	35.03%	33.46%	*P* = 0.40
Gensini score	28.43±22.12	27.66±21.78	*P* = 0.16

Data are shown as mean +/- standard deviation (SD) for quantitative variables and % for qualitative variables.

CAD: coronary artery disease, HDL-c: high density lipoprotein cholesterol levels; LDL-c, low density lipoprotein cholesterol levels, LVSD: left ventricle systolic dysfunction, LVEF: left ventricle eject fraction.

*Age at the first diagnosis of the disease for CAD cases and age at enrollment for CAD controls.

^†^ *P* values for comparison of means for quantitative data with a student t-test, and for comparison of distribution of qualitative variables between CAD patients with LVSD and CAD patients with normal LVEF with a Chi-square test.

**Table 3:** Echocardiographic features in CAD patients with LVSD and CAD patients with normal LVEF.

Ecocardiographic Traits	Males			Females			Combined cohort		
	CAD with LVSD (n = 259)	CAD with normal LVEF (n = 634)	*P*	CAD with LVSD (n = 172)	CAD with normal LVEF (n = 412)	*P*	CAD with LVSD (n = 431)	CAD with normal LVEF (n = 1046)	*P*
Left atrial size (mm)	40.8±4.7	32.3±6.4	8.48×10^–5^	34.8±5.5	32.2±6.6	2.34×10^–3^	38.4±6.1	32.3±6.5	1.17×10^–6^
LV diastolic dimensions (mm)	58.4±5.1	51.7±4.9	6.21×10^–4^	49.4±6.1	48.1±4.1	0.02	54.8±5.8	50.3±5.9	8.82×10^–5^
Interventricular septal thickness (mm)	10.2±3.1	10.7±4.0	0.48	10.0±2.8	10.5±3.7	0.45	10.1±2.9	10.6±3.9	0.30
Aortic root (mm)	39.3±3.9	36.0±3.8	1.23×10^–3^	33.2±5.5	30.9±5.1	0.08	36.9±6.0	34.0±5.9	2.51×10^–4^
LV thickness of the posterior wall (mm)	10.1±3.4	10.2±3.1	0.88	9.8±3.5	10.0±3.7	0.70	10.0±3.4	10.1±3.5	0.66
LVEF (%)	34.1±4.8	57.3±4.1	3.38×10^–8^	33.0±4.6	57.8±3.7	3.38×10^–7^	33.7±4.7	57.5±3.9	5.56×10^–11^

Data are shown as mean +/- standard deviation (SD) for quantitative variables

CAD: coronary artery disease, LV: left ventricle, LVSD: left ventricle systolic dysfunction, LVEF: left ventricle eject fraction.

Statistical power analyses indicated that our study populations provided a sufficient power to detect an association of rs9943582 with CAD (98% power) or LVSD (82% power) assuming an odds ratio (OR) of 1.3 and an alpha level of 0.05.

### Lack of association between SNP rs9943582 and CAD in a GeneID Chinese Han population

Distributions of rs9943582 genotypes did not deviate from the Hardy–Weinberg equilibrium in 1,022 CAD controls (*P* = 0.42). We did not detect any significant association between SNP rs9943582 and the risk of CAD in the GeneID population (*P-abs* = 0.34; empirical *P* = 0.35) (**Table 4**). The association remained non-significant (*P-adj* = 0.22) after adjusting for covariates of gender, age, smoking, hypertension, diabetes and lipids concentrations (Tch, TG, HDL-c and LDL-c) by multivariate logistic regression (**Table 4**).

**Table 4:** Analysis of Allelic Association of SNP rs9943582with Overall CAD and CAD without LVSD.

Cohort (n, case/control)	Count of Genotype in cases			Count of Genotype in controls			Freq _A(case/control)	Observed*		Adjust^†^		Empirical^‡^	Corrected^‖^
								*P-obs*	*OR (95%CI)*	*P-adj*	*OR (95%CI)*	*P-emp*	*P-cor*
	AAA	AAG	GGG	AAA	AAG	GGG							
**Overall CAD**													
Entire cohort (1,751/1,022)	148	653	950	74	382	566	0.27/0.26	0.34	1.06 (0.94–1.20)	0.22	1.08 (0.95–1.23)	0.35	-
Male(1,056/608)	80	382	594	49	223	336	0.26/0.26	0.64	0.96 (0.82–1.13)	0.94	0.99 (0.84–1.17)	0.65	0.87
Female (695/414)	68	271	356	25	159	230	0.29/0.25	0.04	1.23 (1.00–1.49)	0.05	1.22 (1.00–1.49)	0.04	0.08
Hypertension (1,030/525)	92	361	577	40	183	302	0.27/0.25	0.28	1.10 (0.93–1.30)	0.23	1.01 (0.84–1.22)	0.30	0.48
Non-hypertension (721/497)	56	292	373	34	199	264	0.28/0.27	0.72	1.03 (0.86–1.24)	0.91	1.11 (0.93–1.32)	0.75	0.92
**CAD with normal LVEF**													
Entire cohort (1046/1022)	76	382	588	74	382	566	0.26/0.26	0.77	0.98 (0.85–1.13)	0.95	0.99 (0.86–1.15)	0.85	-
Male(634/608)	39	231	364	49	223	336	0.24/0.26	0.25	0.90 (0.75–1.08)	0.36	0.92 (0.76–1.11)	0.25	0.44
Female(412/414)	37	151	224	25	159	230	0.27/0.25	0.34	1.11 (0.89–1.39)	0.35	1.11 (0.89–1.39)	0.34	0.56
Hypertension(613/525)	44	218	351	40	183	302	0.25/0.25	0.96	1.00 (0.83–1.22)	0.91	1.01 (0.83–1.23)	1.00	0.99
Non-hypertension(433/497)	32	164	237	34	199	264	0.26/0.27	0.72	0.96 (0.78–1.18)	0.81	0.97 (0.79–1.20)	0.75	0.92

Freq _A: Frequency of A allele. LVSD: left ventricle systolic dysfunction, LVEF: left ventricle eject fraction.

*Uncorrected *P* value and odds ratio (OR) using Chi-square tests with Pearson’s 2×2.

^†^Adjusted *P* value by multivariate logistic regression analysis for potential confounders including age, gender, smoking, hypertension, diabetes mellitus and lipid concentrations (Tch, TG, HDL-c and LDL-c).

^‡^ Empirical P values were calculated using 100,000 Monte Carlo simulations

^‖^Multiple testing was adjusted by Bonferroni correction

When the CAD cases and controls were divided into subgroups by gender or hypertension, the association between rs9943582 and overall CAD remained non-significant (*P*>0.05; **Table 4**).

### SNP rs9943582 was associated with LVSD in CAD patients

When we divided the CAD patients into a CAD sub-group with LVSD (n = 431) and a CAD sub-group with normal LVEF (n = 1,046) (**Table 2**), we observed significant association between rs9943582 and LVSD in the CAD population (**Table 5**). In 431 CAD patients with LVSD, the frequency of minor allele A was 0.34. In 1,046 CAD patients with normal LVEF (>50%), the frequency of allele A was 0.26. The observed allelic *P-obs* for association was 3.19×10^–6^ with an observed OR of 1.50 (95% CI = 1.27–1.78) (**Table 5**). After adjusting for covariates of gender, age, smoking, hypertension, diabetes and lipids concentrations (Tch, TG, HDL-c and LDL-c) by multivariate logistic regression, the association remained significant with *P-adj* = 6.71×10^–5^ and an adjusted OR of 1.43 (95% CI = 1.20–1.70). The empirical *P* value for the association was estimated to be 8.00×10^–6^ (**Table 5**).

**Table 5:** Analysis of Allelic Association of SNP rs9943582 with LVSD among the CAD population.

Cohort(n, CAD patients with LVSD / CAD patients with normal LVEF)	Count of Genotype in CAD patients with LVSD			Count of Genotype in CAD patients with normal LVEF			Freq _A(CAD patients with LVSD / CAD patients with normal LVEF)	Observed*		Adjust^†^		Empirical^‡^	Corrected^‖^
	AA	AG	GG	AA	AG	GG		*P-obs*	*OR (95%CI)*	*P-adj*	*OR (95%CI)*	*P-emp*	*P-cor*
Entire cohort (431/1046)	52	189	190	76	382	588	0.34/0.26	3.19×10^–6^	1.50 (1.27–1.78)	6.71×10^–5^	1.43 (1.20–1.70)	8.00×10^–6^	-
Male (259/634)	28	98	133	39	231	364	0.30/0.24	0.02	1.31 (1.05–1.65)	0.02	1.32 (1.05–1.66)	0.02	0.04
Female (172/412)	24	91	57	37	151	224	0.40/0.27	1.05×10^–5^	1.80 (1.38–2.35)	4.85×10^–6^	1.87 (1.43–2.44)	1.63×10^–5^	2.10×10^–5^
Hypertension (263/613)	31	109	123	44	218	351	0.33/0.25	1.18×10^–3^	1.45 (1.16–1.81)	2.33×10^–3^	1.48 (1.18–1.86)	1.34×10^–5^	2.36×10^–3^
Non-hypertension (168/433)	21	80	67	32	164	237	0.36/0.26	1.20×10^–3^	1.60 (1.22–2.09)	4.98×10^–6^	1.63 (1.24–2.14)	1.43×10^–5^	2.40×10^–3^

Freq _A: Frequency of A allele. LVSD: left ventricle systolic dysfunction, LVEF: left ventricle eject fraction.

*Uncorrected *P* value and odds ratio (OR) using Chi-square tests with Pearson’s 2×2.

^†^Adjusted *P* value by multivariate logistic regression analysis for potential confounders including age, gender, smoking, hypertension, diabetes mellitus and lipid concentrations (Tch, TG, HDL-c and LDL-c).

^‡^ Empirical P values were calculated using 100,000 Monte Carlo simulations

^‖^Multiple testing was adjusted by Bonferroni correction

Considering that sex and hypertension affect the outcomes of heart failure associated with CAD, we divided the study population into subgroups, including the male group, the female group, a group with hypertension or a group without hypertension. SNP rs9943582 showed more significant association with LVSD in female CAD patients than in the male CAD group (**Table 5**). In the male CAD group contained 259 LVSD cases and 634 controls with normal LVEF, *P-obs* was 0.02 and *P-adj* was 0.04. In the female CAD group contained 172 LVSD cases and 412 controls with normal LVEF, *P-obs* was 1.05×10^–5^ and *P-adj* was 1.63×10^–5^. SNP rs9943582 showed more significant association with LVSD in CAD patients without hypertension than in the CAD group with hypertension (**Table 5**). In the CAD group with hypertension (263 LVSD cases versus 613 controls with normal LVEF), *P-obs* was 1.18×10^–3^ and *P-adj* was 2.33×10^–3^. In the CAD group without hypertension (168 LVSD cases versus 433 controls with normal LVEF), *P-obs* was 1.20×10^–3^ and *P-adj* was 4.98×10^–6^ (**Table 5**).

We compared the homogeneity of ORs between different sub-groups to analyze whether rs9943582 interacted with gender or hypertension using a Breslow-Day test, but detect no significant differences between subgroups divided by gender (observed OR = 1.31, 95% CI from 1.05–1.65 in male and observed OR = 1.80, 95% CI from 1.38 to 2.35 in female, *P* = 0.07) or by hypertension(observed OR = 1.45, 95% CI from 1.16 to 1.81 in hypertension and observed OR = 1.60, 95% CI from 1.22 to 2.09 in non-hypertension, *P* = 0.59) (**Table 5**).

### Assessment of association between rs9943582 and echocardiographic parameters in CAD patients

Using linear regression, we assessed the association of rs9943582 with echocardiographic parameters. In the CAD patients with LVSD, rs9943582 showed significant association with LV diastolic internal dimension (effect size: increased 1.67±0.43 mm per risk A allele, *P* = 1.15×10^–4^), left atrium size (effect size: increased 2.12±0.61 mm per risk A allele, *P* = 9.56×10^–4^) and LVEF (effect size: decreased 2.59±0.32 percent per risk A allele, *P* = 7.50×10^–15^), but not with thicknesses of the interventricular septum (*P* = 0.33), LV thickness of the posterior wall (*P* = 0.61) and the diameter of the aortic root (*P* = 0.61) (**Table 6**).

**Table 6:** Analysis of association of SNP rs9943582 with echocardiographic parameters.

Cohort (n)	LV diastolic dimensions (mm)		Left atrial size (mm)		Interventricular septal thickness (mm)		Aortic root (mm)		LV thickness of the posterior wall (mm)		LVEF (%)	
	Effect size (SE)	*P* value	Effect size (SE)	*P* value	Effect size (SE)	*P* value	Effect size (SE)	*P* value	Effect size (SE)	*P* value	Effect size (SE)	*P* value
**CAD with LVSD**												
Entire cohort (n = 431)	1.67 (0.43)	1.15×10^–4^	2.12 (0.61)	9.56×10^–4^	0.21 (0.22)	0.33	0.21 (0.41)	0.61	0.28(0.46)	0.61	–2.59 (0.32)	7.50×10^–15^
Male (n = 259)	1.27 (0.49)	8.98×10^–3^	1.73 (0.52)	1.18×10^–3^	0.27 (0.20)	0.47	0.02 (0.57)	0.97	0.30(0.51)	0.87	–2.77 (0.42)	2.13×10^–10^
Female (n = 172)	2.41 (0.77)	2.21×10^–3^	2.48 (0.86)	4.88×10^–3^	0.15 (0.36)	0.68	0.26 (0.57)	0.65	0.34(0.68)	0.42	–2.31 (0.52)	1.51×10^–5^
Hypertension (n = 263)	2.00 (0.58)	1.14×10^–4^	2.00 (0.60)	1.34×10^–3^	0.39 (0.29)	0.18	0.74 (0.54)	0.17	0.17(0.45)	0.65	–2.71 (0.39)	4.69×10^–11^
Non-hypertension (n = 168)	1.16 (0.57)	0.02	2.77 (0.90)	3.15×10^–4^	–0.11 (0.34)	0.74	–0.73 (0.64)	0.25	0.40(0.49)	0.20	–2.48 (0.57)	6.19×10^–6^
**CAD with normal LVEF**												
Entire cohort (n = 1,046)	0.64 (0.29)	0.03	–0.11 (0.32)	0.72	–0.35 (0.20)	0.07	0.39 (0.29)	0.18	0.33(0.48)	0.55	–1.17 (0.19)	1.41×10^–9^
Male (n = 634)	0.23 (0.37)	0.54	–0.21 (0.4)	0.54	–0.20 (0.27)	0.46	0.20 (0.31)	0.36	0.32(0.66)	0.89	–0.77 (0.28)	7.02×10^–3^
Female (n = 412)	1.20 (0.48)	0.01	0.05 (0.51)	0.92	–0.52 (0.28)	0.07	0.40 (0.35)	0.32	0.35(0.60)	0.70	–1.49 (0.26)	1.75×10^–8^
Hypertension (n = 613)	1.32 (0.47)	5.12×10^–3^	0.14 (0.42)	0.97	–0.44 (0.26)	0.09	0.46 (0.38)	0.23	0.33(0.45)	0.30	–1.46 (0.24)	1.13×10^–9^
Non-hypertension (n = 433)	0.19 (0.38)	0.61	–0.37 (0.5)	0.48	–0.18 (0.30)	0.55	0.32 (0.44)	0.48	0.33(0.54)	0.68	–0.77 (0.32)	0.02

CAD: coronary artery disease, LV: left ventricle, LVSD: left ventricle systolic dysfunction, LVEF: left ventricle eject fraction.

In the CAD patients with normal LVEF, the association was observed between rs9943582 and LV diastolic internal dimension (effect size: increased 0.64 ±0.29 mm per risk allele A, *P* = 0.03) and LVEF (decreased 1.17±0.19 percent per risk allele A, *P* = 1.41×10^–9^), but not with other echocardiographic parameters (**Table 6**).

We also divided both LVSD and normal LVEF groups into subgroups by gender and hypertension and performed association analysis between rs9943582 and echocardiographic parameters in different groups. In CAD patients with LVSD, significant association was found between rs9943582 and LV diastolic internal dimension, left atrium size and LVEF the in male, female, hypertension and non-hypertension subgroups. In CAD patients with normal ventricular function, significant association was found between rs9943582 and LV diastolic internal dimension only observed in the female group and in CAD patients with hypertension (**Table 6**). For LVEF, significant association was found in all sub-groups (**Table 6**).

### Genotypic association between rs9943582 and CAD or LVSD

Genotypic association between rs9943582 and CAD or LVSD was performed, and the results are shown in **Table 7**. The association of rs9943582 with overall CAD or CAD with normal LVEF was not significant under all three different models (P>0.05) (**Table 7**).

**Table 7:** Analysis of Genotypic Association of SNP rs9943582 with overall CAD, CAD without LVSD, and with LVSD among CAD patients.

Cohorts (n, cases *vs*. controls)	Model	*P_obs* *	*OR (95%CI)*	*P_adj* ^†^	*OR(95%CI)*	*P_emp* ^‡^
All CAD(1,751 *vs*. 1,022)	Dominant	0.57	1.04 (0.90–1.22)	0.35	1.08 (0.92–1.27)	0.58
	Recessive	0.26	1.18 (0.89–1.58)	0.27	1.19 (0.88–1.60)	0.28
	Additive	0.51	n.a	0.23	1.14 (0.97–1.31)	0.51
CAD with normal LVEF(1,046 *vs*. 1,022)	Dominant	0.70	0.97 (0.81–1.15)	0.88	0.99 (0.82–1.18)	0.72
	Recessive	0.98	1.00 (0.72–1.40)	0.92	1.02 (0.72–1.43)	1.00
	Additive	0.92	n.a	0.94	1.00 (0.86–1.15)	0.92
CAD with LVSD (431 *vs*. 1,046)	Dominant	8.98×10^–4^	1.48 (1.15–1.81)	1.80×10^–5^	1.55 (1.23–1.95)	4.66×10^–4^
	Recessive	0.02	1.56 (1.07–2.29)	0.02	1.59 (1.08–2.34)	0.03
	Additive	1.22×10^–3^	n.a	8.98×10^–5^	1.42 (1.19–1.69)	2.12×10^–3^

*P-obs*: *P* value observed, *P-adj*: *P* value with adjustment, *OR*: odds ratio, n.a: no data.

*Uncorrected *P* value and odds ratio (OR) using Chi-square tests with Pearson’s 2×2

^†^Adjusted *P* value by multivariate logistic regression analysis for potential confounders including age, gender, smoking, hypertension, diabetes mellitus and lipid concentrations (Tch, TG, HDL-c and LDL-c).

^‡^ Empirical P values were obtained by performing 100,000 Monte–Carlo simulations.

In the population with 431 CAD patients with LVSD and 1,046 CAD with normal LVEF, significant association was found between SNP rs9943582 and LVSD under both dominant and additive models with *P-obs* of 8.98x10^-4^ and 1.22×10^–3^, respectively (**Table 7**). The association remained significant after adjusting for covariates of age, gender, smoking, hypertension, diabetes mellitus and lipid concentrations with *P-adj* of 1.80x10^-5^ and 8.98x10^-5^, respectively (**Table 7**).

## Discussion

Studies in animal models revealed that the apelin /APJ pathway was important in maintaining the contractile or overall function of the heart. Studies showed that in an isoproterenol-induced model of heart failure, LVSD was partially rescued by co-administration of apelin [12]. More interestingly, acute administration of apelin in patients with chronic heart failure could increase cardiac output and left ventricular performance [24]. These findings demonstrated the important roles of the apelin-APJ pathway in the pathogenesis of heart failure in animal models. In this study, we demonstrated significant association between the *APLNR* gene encoding the apelin receptor and LVSD in humans. We found that the rs9943582 variant in the 5’-untranslated region of *APLNR* conferred a significant risk of LVSD among CAD patients (**Table 5 and Table 6**). A previous study showed that rs9943582 regulated *APLNR* transcription by affecting the binding of Sp1 transcription factor to the *APLNR* promoter, and that the A allele of rs9943582 had lower binding affinity to Sp1 and decreased transcriptional activity than the G allele [25]. Others also showed that Sp1 played a major role in activation of both the TATA-less promoter of *APLNR* and the *Apelin* gene [25,26]. One possible mechanism by which rs9943582 increases risk to LVSD may be attributed to reduced transcription of *APLNR* in myocardium under ischemia/hypoxia injury. Carriers of the G allele, which have more transcriptional activity, may response more effectively to ischemia than carriers with the A allele, and receive more protection from myocardial dysfunction.

The apelin or APJ receptor is a G-protein-coupled receptor (GPCR) with seven transmembrane domains, and has sequence homology with the angiotensin II type 1 receptor (AT1) [10,11]. The only known endogenous ligand of the APJ receptor has been identified as apelin. Apelin is significantly conserved among species and highly expressed in the cardiovascular system as the APJ receptor [27]. In the process of heart failure, the expression levels of apelin and the APJ receptor underwent down-regulation in end-stage failing human hearts. *In vivo* animal modeling or *in vitro* studies showed that expression of endogenous apelin and the APJ receptor was increased immediately after the myocardium was under hypoxia stress, and this up-regulation was confirmed to have a protective effect on the cardiomyocytes from apoptosis or injury [28,29,30]. Increasing evidence points to direct interactions between the apelin-APJ system and the renin–angiotensin system at both molecular and transcriptional levels, and a reciprocal counter-regulatory role for apelin-APJ in relation to the renin–angiotensin system [14,31,32]. Up-regulation of apelin and the APJ receptor under myocardial hypoxia stress may have an inhibitory effect on the renin–angiotensin system and may ameliorate the harmful effect of the AT1 activation [33]. Thus, up-regulation of APJ and apelin in the early stage of heart failure after ischemia may confer a potent protective effect on cardiac contractility and modulate systemic vascular resistance to antagonize the injury of ischemia or hypoxia.

Several studies also showed that the APJ receptor and apelin were associated with atherosclerosis [32,34]. However, in our case-control association analysis, we did not detect any allelic or genotypic association between rs9943582 and CAD in the Chinese Han GeneID population, although the study population provided sufficient statistical power. Our result is consistent with the study by Hinohara et al, which also showed negative association between rs9943582 and CAD in Japanese and Korean populations [35].

In conclusion, for the first time we show that the allele A of rs9943582 (-154G/A) in the *APLNR* gene was associated significantly with left ventricle systolic dysfunction in patients with CAD. These results suggest that the apelin/APJ system is involved in the pathogenesis of heart failure in humans, and provide important insights into the genetic basis and biological pathways for heart failure. Future comprehensive genetic analysis by genotyping and analyzing more SNPs in the *APLNR* gene and other genes in the apelin/APJ pathway as well as haplotype analysis may further define the role of the apelin/APJ pathway in the pathology of heart failure.
